# Effectiveness of Hospital Ambulance Utilization in a Community-Based Integrated Care System

**DOI:** 10.7759/cureus.83754

**Published:** 2025-05-08

**Authors:** Hiroshi Kato, Yutaka Seki, Sumiyo Kitte, Chikara Kinoshita, Hiroshi Mori, Masami Yokoyama, Shotaro Kaneko, Naoki Nakamura, Kunihiro Mashiko

**Affiliations:** 1 Trauma and Critical Care, Medical Corporation Eiseikai Association, Minami-Tama Hospital, Tokyo, JPN; 2 Sports Medical Science, Kokushikan University, Tokyo, JPN; 3 Cardiology, Medical Corporation Eiseikai Association, Minami-Tama Hospital, Tokyo, JPN; 4 Nursing, Medical Corporation Eiseikai Association, Minami-Tama Hospital, Tokyo, JPN; 5 Emergency Medicine, Medical Corporation Eiseikai Association, Minami-Tama Hospital, Tokyo, JPN; 6 Emergency Medical Response, Medical Corporation Eiseikai Association, Minami-Tama Hospital, Tokyo, JPN

**Keywords:** community-based integrated care, emergency transport, geriatric emergency care, hospital ambulance, interhospital transfer

## Abstract

Background and objectives

In recent years, the number of emergency transports for older patients has significantly increased, raising concerns about the strain on emergency medical services. This study aimed to assess whether the use of hospital ambulances can enhance collaboration between home medical care and hospitals while reducing the reliance on fire department ambulances for interhospital transfers.

Methods

This retrospective observational study analyzed 3,983 hospital ambulance dispatches from December 1, 2014, to March 31, 2024. We examined the annual dispatch volume, reasons for dispatch requests, types of transportation, and receiving medical institutions. We then compared the acceptance of ambulance transport and the frequency of interhospital transfers performed by hospital ambulances versus fire department ambulances at chronic care hospitals within the city.

Results

The number of ambulance calls increased annually until 2020 but began to decline in 2021 following the introduction of restrictions on transporting patients with COVID-19. Fractures and orthopedic disorders were the most common reasons for calls (1,040 cases, 26.1%). Interhospital transfer was the most frequent type of transport (1,514 cases, 38.0%), followed by transport from home to the hospital (1,096 cases, 27.5%). Overall, 2,179 patients (54.7%) were transferred to chronic care hospitals, with 3,752 (94.2%) remaining within the city. Regarding the number of ambulances accepted by chronic care hospitals and interhospital transfers, an increase in hospital ambulance utilization and a decrease in fire department ambulance utilization were observed until 2021 and 2020, respectively; however, these trends reversed thereafter.

Conclusion

The use of hospital ambulances facilitates patient transfers to chronic care hospitals and alleviates the burden on fire department ambulances, particularly for interhospital transfers. Hospital ambulances could serve as pivotal tools for reinforcing community-based care while optimizing the use of limited emergency resources.

## Introduction

In Japan, the number of emergency ambulance dispatches by municipal fire departments has been increasing annually. The Fire and Disaster Management Agency (FDMA) reported 7,638,558 nationwide emergency dispatches in 2024 (a 5.7% increase from the previous year), transporting 6,641,420 patients (also a 5.7% increase), both record highs [1]. Rapid population aging is a key factor responsible for this increase [2,3]. The number of older adults (aged ≥65 years) transported by ambulance reached 4,093,552 in 2024 (a 6.0% increase from the previous year), accounting for 61.6% of all patients transported, which previously accounted for 41.4% in 2003 [1]. Additionally, interhospital transfers, often not highly urgent, have been increasing, reaching 556,367 in 2024 (a 3.5% increase year-on-year), accounting for 7.3% of all ambulance dispatches [1]. Such lower-acuity interhospital transfers potentially divert emergency resources from critical situations [4-6].

To address the challenges posed by population aging, Japan’s Ministry of Health, Labour and Welfare (MHLW) has promoted the Integrated Community Care System to enable older adults to receive medical treatment and care within their familiar community [7]. However, overcrowded acute care hospitals can lead to more frequent instances of transport over long distances, making it difficult for patients to return to their home area [2,8]. As the population of older adults continues to grow (projected to peak in 2043 at approximately 39.53 million) [9], the demand for emergency transportation is also expected to increase, highlighting the need for comprehensive regional strategies [2,3,10,11].

In Hachioji City, where Minamitama Hospital (hereafter, “Our Hospital”) is located, local stakeholders established the Hachioji City Geriatric Emergency Medical Collaboration Network (known as “Hachikouren”; hereafter, “Hachikouren”) in 2011 to strengthen medical and long-term care coordination [10,12,13]. As a part of this effort, the Hachioji Medical Association launched the Hachioji City Home Care Patient Emergency Transport Support System (Hospital Ambulance Service) in December 2014. This initiative utilized a hospital ambulance owned by our institution to rapidly transfer older home-based patients in need of hospital care to medical facilities in the city [12,13]. Moreover, to further reinforce regional collaboration and alleviate the workload on fire department ambulances, our hospital introduced a “community-based interhospital transfer program” using the hospital ambulance.

Although the Japan Medical Association (JMA) called for the broader use of hospital ambulances by regional medical associations in 2016 [14], government-regulated deployment of such vehicles remains limited, and evidence of their long-term effectiveness is lacking.

This study aimed to analyze the nine-year operational record of our hospital’s ambulance program and assess its potential contributions in two areas: fostering enhanced collaboration between home medical care and hospitals (home-hospital collaboration) and expanding transfers to local medical facilities, and reducing the volume of interhospital transfers handled by fire department ambulances.

## Materials and methods

Our hospital ambulance service is operated independently from the hospital ambulance program launched in December 2014 by the Hachioji Medical Association. In addition, a separate transport service for elderly residents of care facilities, commissioned by the Tokyo Metropolitan Government, has been in operation since April 2015. Both services are designed for patients whose primary care physicians have determined that hospital transport and admission are medically necessary.

Our program accepts transport requests from all medical institutions in the region, regardless of their affiliation with our hospital, and primarily targets patients with low-acuity conditions who do not require emergency interventions or specialized advanced care. Since the beginning of the service, we have provided bidirectional interhospital transport, both from our hospital to other facilities and from other hospitals to our own. In October 2017, the program was further expanded to include transfers between external hospitals not involving our hospital.

Dispatch requests are accepted only after the referring physician has secured a bed at the receiving hospital in advance. Initially, the service was limited to weekday daytime hours (9:00 AM to 5:00 PM). Weekend daytime service was added in October 2017, and by August 2018, the program had transitioned to a fully operational 24-hour, year-round system.

The service operates with a single ambulance, staffed by a team of two hospital-based emergency medical technicians and one nurse. The vehicle is equipped with a patient monitor, oxygen supply system, emergency medications, medical equipment, and a stretcher. Patients incur no transportation costs, as the service is provided free of charge by the hospital.

This was a retrospective observational study of all hospital ambulance dispatches (n = 3,983) from December 1, 2014, to March 31, 2024. These ambulance dispatches included two categories of transport: one operated under the hospital ambulance program initiated by the Hachioji Medical Association, and the other provided independently by our hospital, comprising facility-based and interhospital transfers. Of these, 2,764 (69.4%) were interhospital transfers. Dispatch data were obtained from Excel files created for each case, and the following six variables were analyzed: (1) annual dispatch volume, (2) reasons for dispatch requests, (3) types of transport, (4) receiving medical institutions, (5) number of transports to chronic care hospitals in Hachioji City by Hachioji Fire Department ambulances (hereafter, “fire department ambulances”) and hospital ambulances, and (6) number of interhospital transfers carried out by hospital ambulances and fire department ambulances.

Data on transport types and receiving medical institutions were also compared with those in our previous report, covering the period up to December 31, 2019 [15]. Interhospital transport was classified into three types based on direction. Upward transfers refer to those from chronic care hospitals to secondary emergency or designated emergency hospitals, or from these to advanced critical care centers. Horizontal transfers included movements between secondary emergency hospitals or between designated emergency hospitals. Downward transfers refer to transfers from advanced critical care centers to secondary or designated emergency hospitals or chronic care hospitals, or from secondary or designated emergency hospitals to chronic care hospitals. Receiving facilities were categorized as "acute care hospitals" (advanced critical care centers, secondary emergency hospitals, and designated emergency hospitals) and "chronic care hospitals," which included long-term care hospitals, rehabilitation hospitals, psychiatric hospitals, and other facilities that do not routinely provide acute emergency care. Data on fire department ambulance transport to chronic care hospitals in Hachioji City (from January 1, 2013, to December 31, 2023) and interhospital transfers by fire department ambulances (from January 1, 2014, to December 31, 2023) were obtained from the Tokyo Fire Department’s Hachioji Fire Station. All data were compiled and organized using Microsoft Excel (Microsoft Corp., Redmond, WA), and results are expressed as frequencies and percentages. Given that the primary objective of this study was descriptive, i.e., to capture the overall situation, no inferential statistical tests (e.g., t-test or chi-square test) were performed.

Ethical considerations

This study was approved by the institutional ethics committee of our hospital (Approval No. 2024-Ack-10). The study was conducted in accordance with the Declaration of Helsinki and relevant ethical guidelines. Patient confidentiality and anonymity were maintained to protect their personal information. The requirement for individual informed consent was waived because of the retrospective nature of this study; however, we ensured transparency and equity in the study design.

## Results

Among the 3,983 cases, the patient age ranged from 0 to 104 years (mean, 77.2 years), with 1,897 (47.6%) men and 2,086 (52.4%) women. During the study period, no cases were reported in which patient transport was considered difficult due to the severity of the patient's condition. Most dispatches were requested by hospital physicians, accounting for 2,884 (72.4%) cases, followed by clinic physicians with 1,061 (26.6%) cases, and others, such as visiting nurses or care managers, with 38 (1.0%) cases. The dispatch sites were predominantly another hospital, totaling 2,767 (69.5%) cases, followed by a patient’s home with 1,100 (27.6%) cases, and a long-term care facility with 116 (2.9%) cases.

**Figure 1:**
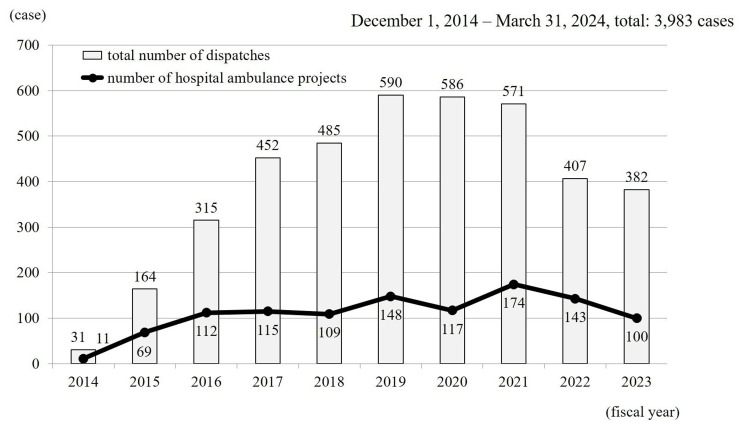
Annual dispatch volume of hospital ambulances The total number of hospital ambulance dispatches, including all uses, peaked at 590 cases in fiscal year (FY) 2019. In contrast, dispatches specifically related to the hospital ambulance project peaked at 174 cases in FY 2021. Both trends have shown a decline since their respective peaks.

Annual dispatches increased each year from project initiation, peaking at 590 in fiscal year (FY) 2019, and then declining thereafter. Hospital ambulance services alone accounted for a cumulative 1,098 (27.6%) dispatches, gradually increasing through FY 2021 (174 at peak) before showing a downward trend (Figure 1).

**Figure 2:**
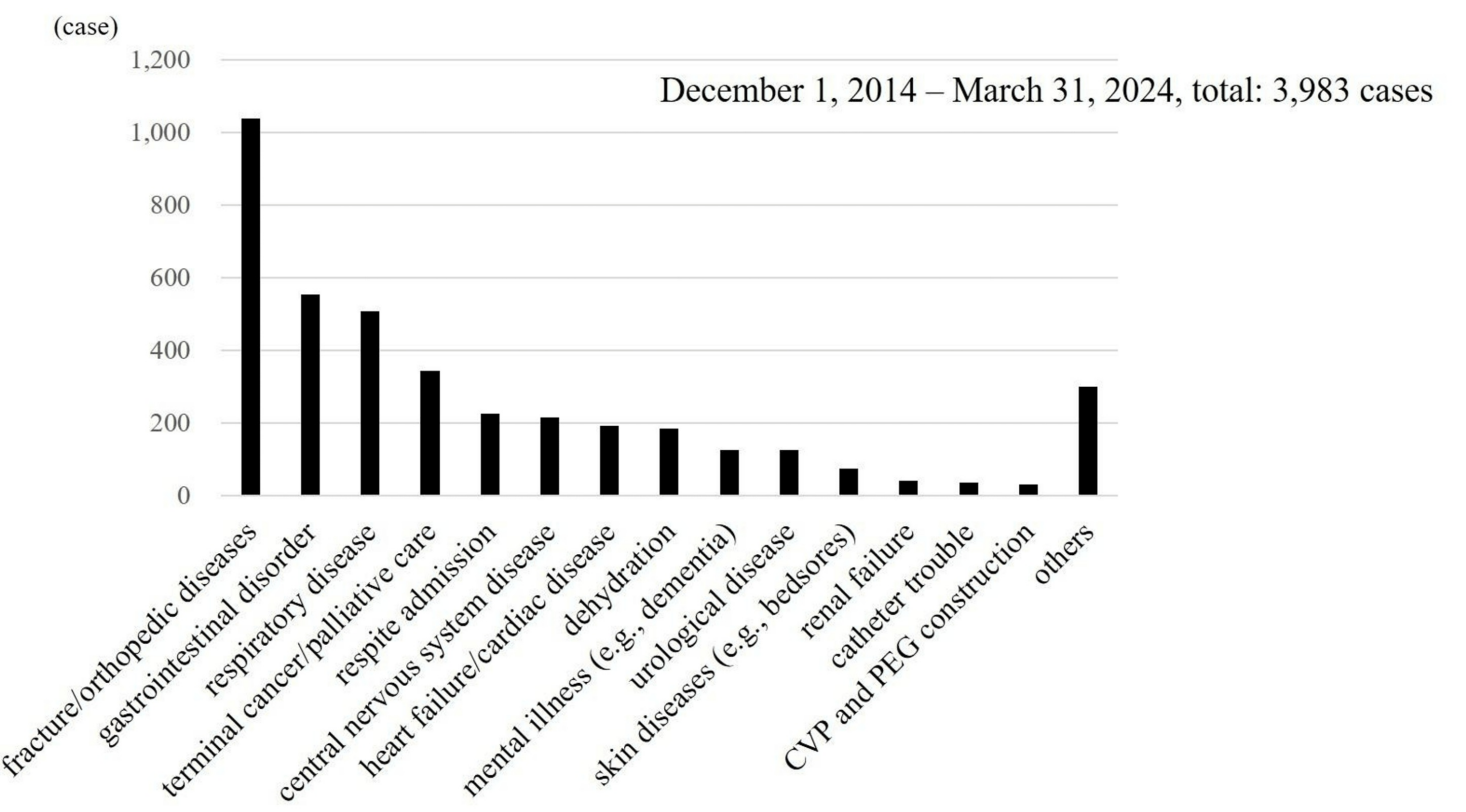
Reasons for requesting hospital ambulance transport Orthopedic conditions were the most common, followed by gastrointestinal and respiratory issues, palliative care needs, and respite admissions. Respite hospitalization refers to admission intended to provide rest or relief for caregivers. CVP, central venous port; PEG, percutaneous endoscopic gastrostomy

The most frequent reason was fractures/orthopedic disorders, accounting for 1,039 (26.1%) cases, followed by gastrointestinal complaints (abdominal pain, vomiting, and diarrhea), respiratory issues (pneumonia and acute exacerbation of chronic respiratory failure), end-stage cancer/palliative care, and respite admissions (Figure 2).

**Figure 3:**
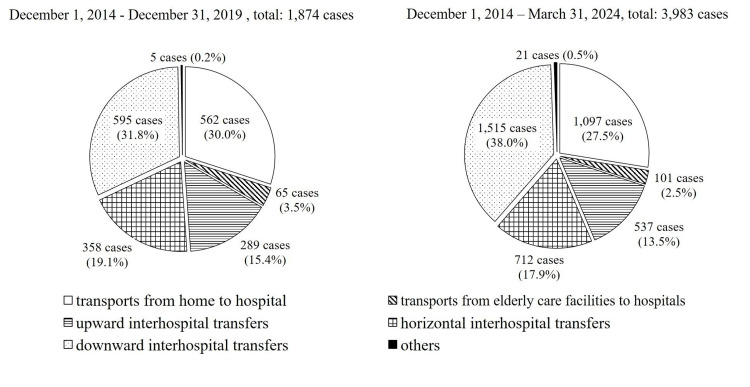
Types of hospital ambulance transport The left chart shows the distribution of transport types through the calendar year 2019 (n = 1,874), and the right chart shows updated totals through FY 2023 (n = 3,983). Percentages in FY 2023 are rounded, totaling 99.9%. Downward inter-hospital transfers were the most common in both periods, with a higher proportion observed in FY 2023.

Between December 1, 2014, and March 31, 2024, the most common transport type was downward interhospital transfers, with 1,515 (38.0%) cases, followed by transports from home to hospital with 1,097 (27.5%) cases, horizontal interhospital transfers with 712 (17.9%) cases, upward interhospital transfers with 537 (13.5%) cases, transports from older patient care facilities to hospitals, with 101 (2.5%) cases, and “other” with 21 (0.5%) cases. Compared with our previous 2019 report [15], the ranking of transport types remained unchanged, although the proportion of downward interhospital transfers increased by 6.2% (from 31.8% to 38.0%) (Figure 3).

Of the 2,764 interhospital transfers, 2,312 (83.6%) were from our hospital to another facility, 251 (9.1%) were from another facility to our hospital, and 201 (7.3%) were from one facility to another without involving our institution. Of the 1,097 transports originating from homes, 421 (38.4%) went directly to acute care hospitals and 676 (61.6%) went to chronic care hospitals.

**Figure 4:**
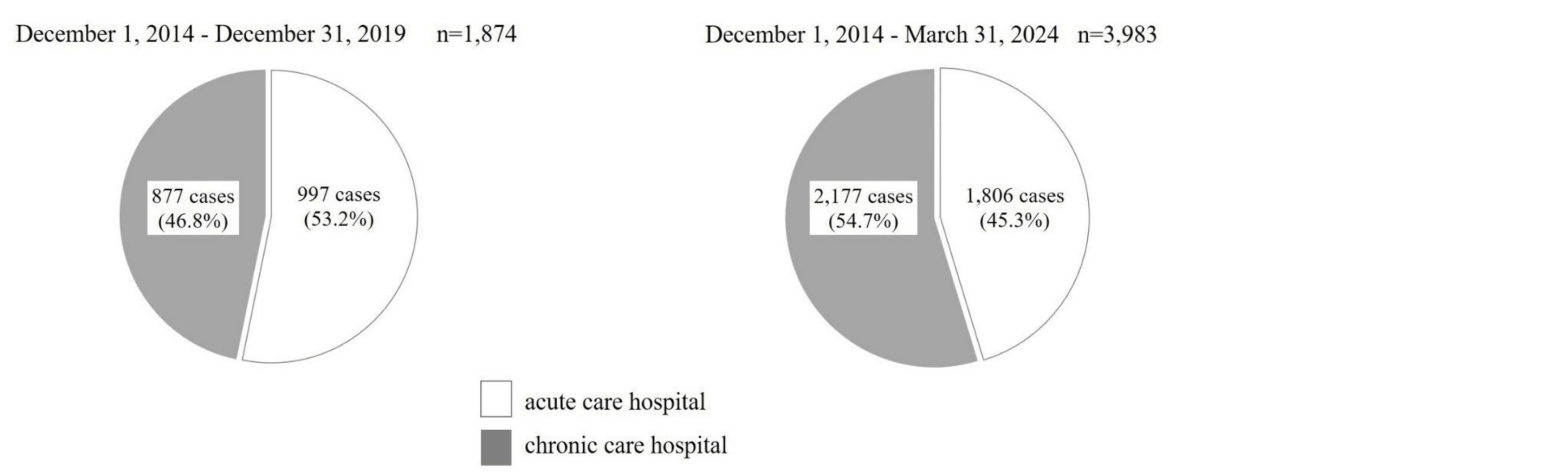
Receiving medical institutions The left chart shows the distribution of transports to acute and chronic care hospitals through the calendar year 2019 (n = 1,874), and the right chart shows updated totals through FY 2023 (n = 3,983). Compared to 2019, the proportion of transports to chronic care hospitals increased in FY 2023.

Among the 1,874 cumulative dispatches as of December 31, 2019, 997 (53.2%) were to acute care hospitals and 877 (46.8%) were to chronic care hospitals [15]. By March 31, 2024, the proportions shifted to 1,806 (45.3%) for acute care hospitals and 2,177 (54.7%) for chronic care hospitals (Figure 4).

Notably, 1,324 (73.3%) of the acute care hospital transports involved eight hospitals within Hachioji City (out of 15), whereas 2,045 (94.2%) of the chronic care hospital transports involved nine hospitals within Hachioji City (out of 14).

**Figure 5:**
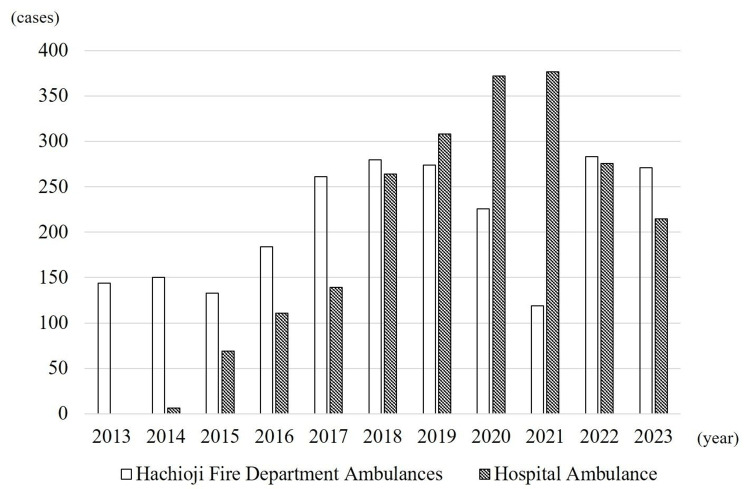
Number of ambulance transports to chronic care hospitals in Hachioji City Until 2021, transports by hospital ambulances steadily increased, while those by fire department ambulances began to decline after 2019. Since 2022, this trend has reversed, with hospital ambulance transports decreasing and fire department ambulance transports increasing.

The number of ambulances transported to chronic care hospitals in Hachioji City increased each year from 144 (100.0% fire department ambulances) in 2013 to 598 in 2020, comprising 226 (37.8%) fire department ambulances and 372 (62.2%) hospital ambulances (Figure 5). Between 2019 and 2021, chronic care transport by fire department ambulances declined, whereas that by hospital ambulances rose sharply, peaking at 377 (76.0% of the total 496) in 2021. However, after 2022, the number of hospital ambulance transports decreased, whereas that of fire department ambulance transports increased again to over 250 per year (Figure 5).

**Figure 6:**
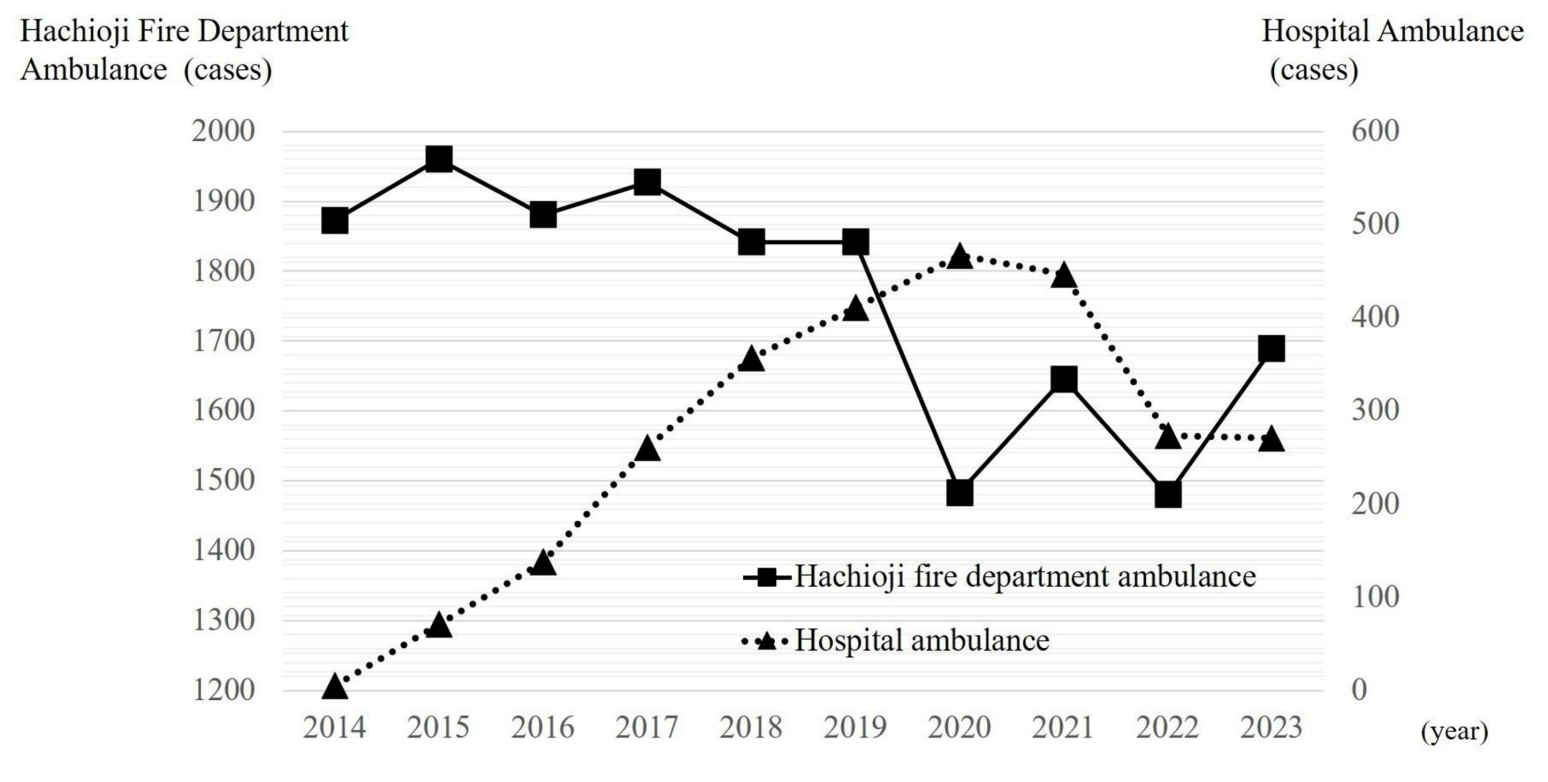
Number of interhospital transfers From 2014 onward, inter-hospital transfers by hospital ambulances showed an increasing trend, while those by fire department ambulances declined. Since 2021, hospital ambulance transfers have decreased, whereas fire department ambulance transfers stabilized and increased again in 2023.

From 2014, interhospital transfers by hospital ambulances steadily increased, whereas those by fire department ambulances decreased. In 2020, the number of interhospital transfers by hospital ambulances reached a record high of 467, representing an increase of 56 cases (13.6%) from the previous year, whereas the number transported by fire department ambulances decreased sharply to 1,483, a reduction of 359 cases (19.5%) from the previous year. However, after 2021, hospital ambulance transfers declined, and fire department transfers stopped decreasing and even showed a rebound by 2023 (Figure 6).

## Discussion

In this nine-year review of hospital ambulance utilization, dispatches showed a marked rise during the initial six years, with roughly one-quarter attributable to the hospital ambulance service. Over 70% and 90% of acute and chronic care hospital transport, respectively, involved facilities located within Hachioji City, implying that hospital ambulances served as an effective safety net, supporting the region’s integrated community care system.

Notably, transfers to chronic care hospitals have recently surpassed those to acute care hospitals. Although our data through 2019 showed a greater number of transports to acute care hospitals overall, the trend shifted toward chronic care facilities in subsequent years.

Furthermore, much of the overall increase in chronic care hospital admissions was driven by hospital ambulance transports, suggesting that hospital ambulances are effective for enhancing interhospital transfers, improving home medical care-hospital collaboration, and facilitating timely patient flow within the community. Notably, over 60% of transports from patients’ homes were direct to chronic care hospitals, further underscoring the role of hospital ambulances in streamlining transitions of care, particularly for older or lower acuity patients.

One of the key reasons for the increase in ambulance admissions to chronic care hospitals is the strengthened collaboration among stakeholders, including home care providers, acute care hospitals, and chronic care hospitals, who share the challenges of increasing transport difficulties for elderly patients and the rising burden on fire department ambulances. This collaboration has facilitated smoother coordination with receiving hospitals, allowing for advanced bed allocation. Furthermore, accurate clinical information from primary care physicians is directly communicated to chronic care hospital physicians, enabling quicker and more confident decisions about patient acceptance. These factors together may have contributed to the observed increase in admissions.

The most common transport type in this study was “downward interhospital transfers.” Most of the patients included in this study were older adults, and the increasing trend in downward transfers via hospital ambulances may have been influenced by population aging. Specifically, we assume that a significant proportion of patients do not sufficiently recover their activities of daily living (ADLs) after completing acute care treatment, making it difficult for them to travel by private car. As a result, hospital ambulances may represent a more practical option for transport to chronic care hospitals. Additionally, the absence of patient costs for using hospital ambulances may have also contributed to the increase in transfer requests. Delayed transfers out of acute care hospitals can lead to “exit blocks” that prolong hospital stays, which in turn cause “entry blocks” that limit the capacity to cater to new emergency cases [16]. In Hachioji City, the local collaboration network (Hachikouren) actively promoted the early transfer of patients who no longer require specialized acute care [12,13]. Our hospital extended its ambulance services to facilitate transfers not only from our facility but also from other hospitals. The increase in the proportion of downward interhospital transfers compared to that in the 2019 data likely reflects the ongoing success of this broader regional collaboration.

The observed trends in home care patient transfers to chronic care hospitals and early transfers from acute to chronic care hospitals highlight the role of hospital ambulances as a fundamental component of an older people-focused emergency medical system. This system integrates all the medical institutions within the region, including home medical care services. Hospital ambulances play a pivotal role in the efficient functioning of these systems.

In March 2016, Japan’s FDMA and MHLW jointly issued a notification encouraging the use of hospitals or private ambulances for low-acuity interhospital transfers [17]. Following this directive, the number of interhospital transfers handled by the Tokyo Fire Department decreased by 13.8% by 2020 [18]. In Hachioji City, the decrease was even more pronounced at 19.5%. Meanwhile, interhospital transfers through our hospital ambulance grew by 13.6% in 2020, indicating that increased hospital ambulance utilization for less urgent transfers likely contributed to reducing the burden on fire department ambulances. A similar trend was observed in a study conducted at a university hospital in Tokyo, which analyzed 1,368 hospital ambulance cases and reported a reduction in fire department ambulance utilization for interhospital transfers [19]. Thus, deploying hospital ambulances for low-acuity interhospital transfers appears to be a valid strategy to free up public emergency services for more urgent cases.

Despite the benefits of hospital ambulances, several challenges remain with regard to their broader implementation, including government policies, financial support, and workforce training [11,20]. Ito et al. conducted a trial operation of interhospital transfers for low-acuity patients utilizing hospital ambulances staffed with hospital-based emergency medical technicians under online medical control (MC) from the regional MC council. The study reported safe and efficient patient transport in all cases, further supporting the viability of hospital ambulances as an alternative to fire department ambulances [20,21]. Nationwide expansion of such a hospital-based ambulance model is anticipated in the future. Furthermore, our hospital’s ambulance service operates under a unique system in which nurses accompany paramedics on board, unlike standard fire department ambulances. Although this study did not conduct a detailed analysis of difficult transport cases, this staffing configuration may contribute to safer and more effective patient management during transportation. This potential benefit warrants further investigation in future studies.

A notable decline in hospital ambulance dispatches and interhospital transfers was observed after 2021, largely owing to COVID-19-related restrictions. At our hospital, hospital ambulances are not used for patients with confirmed or suspected COVID-19 to minimize the risk of infection and avoid prolonged ambulance downtimes due to decontamination procedures.

Additionally, Hachioji City authorities coordinated the transport of patients with COVID-19 by utilizing designated private ambulances and care taxis rather than hospital ambulances. However, a Tokyo Medical Association Emergency Committee survey found that 485 patients with confirmed or suspected COVID-19 were transported via hospital ambulances between January 1 and December 31, 2021 [11]. The report highlighted that hospital ambulances functioned as a crucial component of the emergency transport infrastructure, particularly amid increased difficulties in hospital selection. Moving forward, the role of hospital ambulances in infectious disease outbreaks requires further examination and strategic planning.

This study had three main limitations. First, we analyzed only the fire department ambulance data from Hachioji City and excluded transportation initiated by municipalities outside the city. Therefore, the actual total number of interhospital and chronic care hospital transfers may be slightly larger than what our study reports. Second, we did not assess the nature of requests for hospital ambulance dispatches or the frequency of declining or overlapping requests in detail. The actual demand for hospital ambulances may have been higher than that suggested by the dispatch data; conversely, some patients transported by our hospital ambulance might have been safely served by private ambulances or care taxis. Given that fire departments and hospital ambulances are finite resources, evaluating and refining policies to ensure appropriate utilization remains essential. Third, this study was conducted in a single city in Japan, and while the findings may provide useful insights for regions or countries with similar emergency transport systems, their generalizability to areas with different systems may be limited.

## Conclusions

This study suggests that establishing a hospital ambulance system within a strengthened regional medical collaboration framework may enhance home medical care-hospital collaboration and increase the number of transfers accepted by local chronic care hospitals. In addition, the findings indicate that hospital ambulances could potentially reduce the number of “downward” interhospital transfers handled by fire department ambulances, which may help alleviate the burden on municipal emergency medical services. However, as this study is retrospective and observational in nature, causal relationships cannot be firmly established, and further prospective studies are warranted to validate these findings. Nevertheless, as Japan’s population continues to age, hospital ambulances may play an important role in reinforcing community-based care and optimizing the use of limited emergency resources.
